# Feasibility study to inform the design of a UK multi-centre randomised controlled trial of prophylactic antibiotics for the prevention of recurrent cellulitis of the leg

**DOI:** 10.1186/1745-6215-8-3

**Published:** 2007-01-26

**Authors:** Kim S Thomas, Neil H Cox, Boki SP Savelyich, Debbie Shipley, Sarah Meredith, Andrew Nunn, Nick Reynolds, Hywel C Williams, on behalf of the UK Dermatology Clinical Trials Network (UK DCTN)

**Affiliations:** 1 Centre of Evidence Based Dermatology, University of Nottingham, Nottingham, UK; 2 Department of Dermatology, Cumberland Infirmary, Carlisle, UK; 3 Department of Dermatology, Bristol Royal Infirmary, Bristol, UK; 4 MRC Clinical Trials Unit, London, UK; 5 Department of Dermatology, University of Newcastle, Newcastle, UK

## Abstract

**Background:**

This paper describes the results of a feasibility study for a randomised controlled trial (RCT).

**Methods:**

Twenty-nine members of the UK Dermatology Clinical Trials Network (UK DCTN) expressed an interest in recruiting for this study. Of these, 17 obtained full ethics and Research & Development (R&D) approval, and 15 successfully recruited patients into the study. A total of 70 participants with a diagnosis of cellulitis of the leg were enrolled over a 5-month period. These participants were largely recruited from medical admissions wards, although some were identified from dermatology, orthopaedic, geriatric and general surgery wards. Data were collected on patient demographics, clinical features and willingness to take part in a future RCT.

**Results:**

Despite being a relatively common condition, cellulitis patients were difficult to locate through our network of UK DCTN clinicians. This was largely because patients were rarely seen by dermatologists, and admissions were not co-ordinated centrally. In addition, the impact of the proposed exclusion criteria was high; only 26 (37%) of those enrolled in the study fulfilled all of the inclusion criteria for the subsequent RCT, and were willing to be randomised to treatment.

Of the 70 participants identified during the study as having cellulitis of the leg (as confirmed by a dermatologist), only 59 (84%) had all 3 of the defining features of: i) erythema, ii) oedema, and iii) warmth with acute pain/tenderness upon examination.

Twenty-two (32%) patients experienced a previous episode of cellulitis within the last 3 years. The median time to recurrence (estimated as the time since the most recent previous attack) was 205 days (95% CI 102 to 308).

Service users were generally supportive of the trial, although several expressed concerns about taking antibiotics for lengthy periods, and felt that multiple morbidity/old age would limit entry into a 3-year study.

**Conclusion:**

This pilot study has been crucial in highlighting some key issues for the conduct of a future RCT. As a result of these findings, changes have been made to i) the planned recruitment strategy, ii) the proposed inclusion criteria and ii) the definition of cellulitis for use in the future trial.

## Background

Cellulitis of the lower leg is an acute, painful and potentially serious infection of the skin and subcutaneous tissue. It is very common and currently accounts for 2–3% of hospital admissions [1]. The average length of inpatient stay is 9 days (Hospital Episode Statistics, Department of Health, 2002–2003), and 25–50% of treated patients suffer further episodes and other morbidity, such as oedema and ulceration when followed-up for a number of years [1,2].

There are numerous risk factors for cellulitis of the lower leg, including previous episodes of cellulitis; leg oedema (especially lymphoedema); toeweb maceration (often caused by tinea pedis); obesity and diabetes [2-4]. Approximately one third of patients have recurrent episodes, at least in part due to the above risk factors.

Existing evidence for the use of prophylactic antibiotics to prevent further episodes is very limited. Two small RCTs hint at possible benefit, but these studies are very small (16 and 40 participants respectively) [5,6]. A large-scale, multi-centre trial evaluating the use of prophylactic antibiotics for the prevention of cellulitis of the leg is planned (PATCH study – **P**rophylactic **A**ntibiotics for the **T**reatment of **C**ellulitis at **H**ome). Two parallel RCTs have now been funded and are being co-ordinated through a network of dermatologists, dermatology nurses and health services researchers with an interest in research into skin disease. However, prior to commencing and in order to inform such a large trial, the feasibility study described below was conducted. This study had the following objectives: i) to inform the design of the RCT by gathering data on demographics, baseline event rates and the impact of eligibility criteria; ii) to test the feasibility of recruiting and running such a trial through the UK DCTN; and iii) to collect service users' views on the design and conduct of the trial.

## Methods

### Design

This feasibility study had three aspects: i) structured interviews with patients admitted to hospital with a diagnosis of cellulitis of the leg; ii) a questionnaire survey of recruiting dermatologists; and iii) a focus group discussion involving 5 patients with a history of recurrent cellulitis. No specific interventions were administered during the study.

### Setting/participants

A total of 29 hospitals throughout the UK and Southern Ireland volunteered to take part in the study. These comprised of 20 teaching hospitals and 9 district general hospitals. Patients were asked to take part in the study if they had been admitted to hospital with a diagnosis of cellulitis of the leg. All patients received standard treatment for their cellulitis according to the treating physician's normal practice.

### Recruitment

Recruitment took place over a 5-month period from November 2004 to March 2005.

All inpatients that had been identified as having cellulitis of the leg were approached by a UK DCTN dermatologist or dermatology nurse during their stay in hospital. Other than a confirmed diagnosis of cellulitis of the leg, no additional inclusion/exclusion criteria were used in order to test the effects of the proposed eligibility criteria for the future RCT. Data were collected on patient demographics, presenting clinical features and previous medical history. At the end of the study period, a short postal survey was completed by the recruiting physician/nurse. This was used to highlight any methodological problems or concerns encountered during the period of the study.

### Involvement of service users

Part of the remit of the pilot study was to capture service users' views on the trial design. Having been given a brief description of the proposed study, participants were asked to comment on their willingness (or otherwise) to take part in a future RCT and to identify possible areas of concern that may limit their involvement.

A focus group of five patients with experience of cellulitis was convened in order to explore these issues more fully. The discussion was audio-taped and fully transcribed for analysis. Prior to discussing the implications of participating in the proposed RCT, members of the focus group were given both written information and an oral presentation, in which the key aspects of the trial were outlined.

### Ethics/R&D

All necessary ethical and R&D approvals were in place prior to commencement of the study. The study was approved by the Metroplitan Multi-Centre Research Ethics Committee (ref: 04/MRE11/26).

All data were entered onto a customised Microsoft^® ^Access database and analysed using SPSS, version 11.5.

## Results

### Recruiting centres

**Figure 1:**
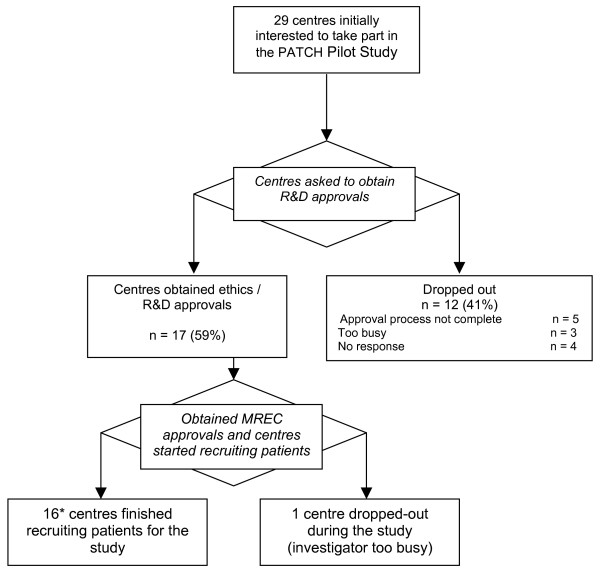
Flow chart of recruiting centres. [* Two centres did not recruit any patient.]

Of the 29 centres that had initially expressed an interest in helping with recruitment for this trial, only 17 (59%) centres had all of the necessary approvals in place ready to enrol patients at the start of the recruitment period (Figure 1). Of these, 15 centres actually recruited participants into the study. The main limiting factors in obtaining the approvals were a lack of time and administrative resource at the recruiting centres.

### Participant recruitment

**Figure 2:**
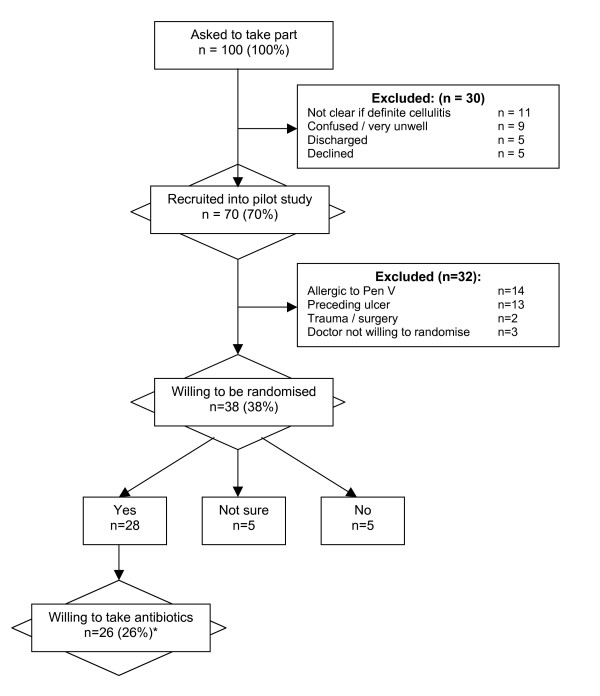
Impact of exclusion criteria on recruitment rates. [* Of the 26 participants who met the inclusion criteria and were willing to take part in the study, 9 had had a recurrence within the last 3 years (6 within the last 12 months).]

A summary of recruitment rates throughout the 5-month recruitment period is presented in Figure 2. In 63% (42/67) of the cases, participants were recruited from general medical wards. Only 12% (8/67) came from dermatology wards. The remainder came from a variety of sources including orthopaedic, geriatric and general surgery wards. A total of 70 participants were recruited from 15 centres. The median number of patients recruited was 3 per centre (min 0, max 12).

### Impact of exclusion criteria

All patients with a diagnosis of cellulitis of the leg were asked to take part in this study. However, for the purposes of the RCT exclusion criteria will apply. Figure 2 presents the likely implications of these exclusion criteria on the overall recruitment rate. Of the 100 participants who were initially identified, only 26 were potentially eligible and willing to take part in the future RCT.

### Participants' characteristics

A summary of participants' characteristics is presented in Table 1. This confirms the importance of known risk factors for cellulitis, such as obesity, diabetes, lymphoedema and toe-web maceration.

**Table 1:** Participant characteristics. [Note: with the exception of BMI, all variables had <3 missing values. For BMI data were missing for 24 cases due to difficulties in measuring the height and weight of patients.]

**Characteristic**	**n (%)**
Male	41 (61)
Female	26 (39)
Age – mean	61 (s.d 16.0)
Age range	22 to 87
Body Mass Index – BMI (kg/m2)	
Underweight (<20)	1 (2)
Normal (20 to 24.9)	11 (24)
Overweight (25 to 29.9)	8 (17)
Obese (>30)	26 (57)
Diabetes	14 (21)
Lymphoedema	11 (16)
Toe-web maceration	22 (32)
DVT prior to acute episode	2 (3)
Pre-existing ulcer	14 (20)
Previous blunt injury or scratch	19 (28)
Post operative or penetrative wound	6 (9)
Known allergy to penicillin	14 (20)
Previous episode (ever)	30 (44)
Previous episode in last 3 years	22 (32)

### Definition of cellulitis

The criteria for defining an eligible case of cellulitis for inclusion in the RCT were to be developed from the results of this pilot study. As presented in Table 2, all of the recruited patients were confirmed to have cellulitis of the leg by a dermatologist or dermatology nurse. Entry criteria based on the presence of i) erythema, ii) oedema, and iii) warmth with acute pain/tenderness results in an eligible population of 59 (84%). By including the additional criteria of constitutional disturbance (flu-like symptoms), this rate falls to 39 (56%). Alternatively, the use of unilateral disease as a defining feature (in combination with i to iii above) results in an eligible population of 55 (79%).

**Table 2:** Defining characteristics of cellulites

**Characteristics**	**n (%)**	
Cellulitis as confirmed by dermatologist/dermatology nurse	70 (100)	
i) Erythema	69 (99)	i, ii, & iii, 59 (84%)
ii) Oedema	63 (90)	
iii) Warmth with acute pain/tenderness	66 (94)	
iv)Constitutional disturbance	43 (62)	iv plus i to iii above 39 (56%)
v) Unilateral disease	63 (90)	v plus i to iii above 55 (79%)

### Previous episodes of cellulitis

**Figure 3:**
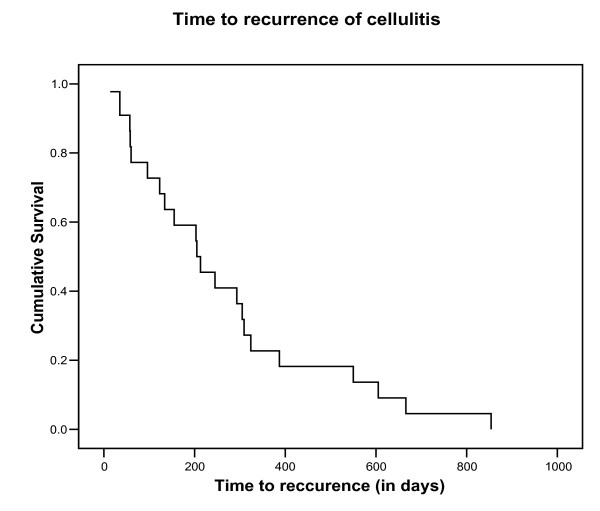
Time to recurrence for patients with a relapse within the last 3 years.

Overall, 44% (30/68) of participants reported having had a previous episode of cellulitis, although only 55% of these (17/30) were admitted to hospital for the treatment of their cellulitis during their most recent previous attack. Seven percent of cases (2/29) received prophylactic antibiotics and 14% (4/29) were prescribed compression bandages. The number of previous episodes of cellulitis ranged from 0 to 6 (median = 1). Based on those with a recurrence within the last 3 years (22/68, 32%), the median time to recurrence was 205 days (95% CI 102 to 308). (Figure 3). For those with a recurrence at any time (n = 30), the median time to recurrence was 293 (95% CI 169 to 417).

### Treatment of the current episode

During the current episode of cellulitis, 45% (31/69) of participants received IV treatment, 44% (30/69) received a combination of IV and oral antibiotics; and 12% (8/69) received oral antibiotics. Where data were available, the duration of treatment was between 7 and 14 days.

### Feedback of investigators

A summary of comments received by investigators is presented in Table 3. The main area of concern relates to the identification of patients (only 42% of investigators felt that this worked well). Similarly, there were concerns about the time required to do the study and the lack of administrative support; particularly in the preparation of ethics applications and institutional approvals.

**Table 3:** Summary of lessons learnt from the feasibility study

**Issue**	**Problem**	**Solution implemented for the RCT**
Definition of cellulitis	• Only 70% of those with cellulitis (as confirmed by a dermatologist) fulfilled the planned inclusion criteria to be used for the confirmation of cellulitis in the RCT.	• Inclusion criteria modified to be "Cellulitis as confirmed by a dermatologist". Individual clinical features will also be reported.
Recruitment	• Considerable difficulties in relying on UKDCTN members to recruit into the study.	• Measures to increase recruitment include: displaying information in relevant clinics; presenting at hospital clinical meetings; recruiting through A&E and acute medical wards; identifying patients through coding departments; and paying for greater administrative support at the recruiting centres.
Definition of recurrent cellulitis	• A definition of recurrent cellulitis for use in the trial was required.	• Recurrent cellulitis is defined as being "at least one previous episode of cellulitis of the leg within the preceding 3 years".
Alternative antibiotic for patients with penicillin allergy	• A surprisingly high proportion reported penicillin allergy (20%). Should an alternative be provided within the trial?	• No. The disadvantages (increased cost, more side effects and requirement for a double dummy) outweighed the recruitment advantage.
Treatment of existing risk factors	• During the trial, dermatologists will be increasingly involved in the care of patients with cellulitis. If this alters the normal clinical practice of the treating physician, this could reduce the recurrence rates seen in the control arm. For example, should the dermatologists recommend treatment of tinea pedis?	• Unethical not to highlight the need for treatment if risk factors are observed. The treating physician will be asked to follow usual practice and risk factors treated on their merit.
Impact of antibiotic resistance	• Concerns were expressed by patients, funding bodies and the ethics committee about the possible impact of long-term antibiotic therapy on microbial resistance.	• A review of the literature suggested that streptococcal infections have remained susceptible to penicillin for over 60 years, despite wide-spread use. There is no evidence to suggest that low-dose penicillin (which is currently used for other conditions, e.g.rheumatic fever) will lead to drug resistance. This fact is discussed at length in the supporting patient information leaflets.

### Feedback of participants

Feedback given during the structured interview highlighted particular areas of concern that might influence a patient's decision to volunteer for a future RCT. These included:

◊ not willing to take tablets/placebo

◊ not willing to take antibiotics (if not necessary for treatment)

◊ worries about resistance to antibiotics

◊ inability to travel for the research

◊ too old for a long term study/long term commitment

◊ worried that could not drink alcohol during the trial

◊ previous bad reactions to antibiotics

◊ would prefer once a day rather than twice a day dosing

◊ concerns relating to co-morbidity.

Results of the focus group showed similar concerns. When the participants were asked to discuss if, hypothetically, they would be happy to take part in the study, it was evident that they required detailed information before being able to make this decision, especially with regard to:

◊ Concerns about taking antibiotics. This included issues such as: drug interactions; GI side effects and building up resistance; whether or not tablets should be taken on an empty stomach and when they should be taken; and the possibility of not being able to drink alcohol for the duration of the study.

◊ What would happen if they experienced another medical problem requiring hospitalisation and/or the use of other antibiotics during the study?

◊ Keeping and using emergency antibiotics for acute onset of an episode of cellulitis – would this be allowed?

◊ Were they allowed to take other medications during the study?

◊ What would happen at the end of the study – could they move onto other antibiotics or receive confirmed prophylaxis?

Interestingly, few issues were raised about taking placebo (despite specific questioning). However, there was a reluctance to stop taking current prophylactic antibiotics, as those who were taking antibiotics in this way felt reassured by their use.

## Discussion

### Main findings

This feasibility study proved invaluable in resolving several crucial design issues for the future RCT. In particular it addressed six key areas:

#### 1) Identification of recruiting centres

Despite an enthusiastic response to the initial call for recruiting centres, over 40% of those who expressed an interest in the trial were unable to obtain the necessary ethics and R&D approvals within the time window available. The increasing paperwork surrounding the conduct of clinical trials following the introduction of the EU Clinical Trials Directive, and the implementation of the Research Governance Framework in the UK means that much of this process is not optional. The set-up of this feasibility study coincided with the introduction of the new electronic form for ethics applications, and there were inevitable teething problems associated with this development. In addition, at the time of this research, many hospital Trusts were not accepting the standard R&D application form, thus making the R&D approval process even more difficult. Considerable efforts have since been made to streamline the approval process in the UK. Developments such as the on-line standard R&D form and the plans to subsume site specific ethical review within the local R&D approval process are to be welcomed.

#### 2) Recruitment of participants

Recruitment for the pilot study was at approximately 1 patient per centre per month (15 per month in total). However, the impact of applying exclusion criteria for the RCT meant that this figure was dramatically reduced. Of the 100 participants originally approached about the study, only 26 (26%) fulfilled the eligibility criteria and were willing to be randomised to treatment. In addition, the impact of a more stringent definition of cellulitis means that this figure could be reduced still further.

Investigators also reported that locating patients was difficult. This has worrying implications for an RCT involving patients with cellulitis who are recruited by dermatologists in a secondary care setting. It is important to understand why identifying patients with such a common condition could be so difficult. Feedback from the recruiting centres suggests that one of the most important limiting factors for recruitment was that patients were not routinely treated by dermatologists. Patients were seen by many different disciplines (general medicine, infection control, emergency medicine, geriatrics); were admitted through various routes; and were often moved or discharged at short notice. In addition, 6 centres established a new system of community-based care during the period of the pilot study. This meant that fewer patients were treated for cellulitis in hospital and those that were admitted were more likely to have multiple co-morbidities, or to have cognitive impairment, making them unsuitable for inclusion in a trial. It is likely that other hospitals will adopt similar policies in the future.

The solution to different routes of admission for recruitment seems to vary from hospital to hospital: providing greater administrative support; posters on relevant wards; identification of a link person (who may be a registrar or research nurse, rather than necessarily the consultant); tracking admissions from admission ward/casualty department records; identification from clinical coding records; and 'advertising' by a specific discussion of the study at a postgraduate meeting or reminders at directorate meetings are all possible methods to increase recruitment.

#### 3) Inclusion/exclusion criteria

Inclusion into the RCT will be based on a diagnosis of cellulitis of the leg. However, cellulitis is a difficult diagnosis to confirm by bacteriology and no guidelines exist relating to clinical criteria for entry into trials. Obviously for any future trials a balance must be struck between the specificity and sensitivity of the chosen inclusion criteria. Similarly, the impact of possible exclusion criteria was high. In particular, 14 (20%) of those recruited into the pilot were allergic to penicillin, and a further 13 (19%) had a preceding ulcer, both of which are planned exclusion criteria for the future RCT.

#### 4) Primary outcome – time to recurrence

Data relating to time to recurrence suggest that the majority of patients have a subsequent episode within a time frame suitable for capture within the duration of a trial, (indeed 75% occurred within 12 months of the previous episode). In the 22 (32%) of participants who had experienced a previous attack, the median time to recurrence was 205 days (95% CI 102 to 308). This supports the need for medium-term therapy for the 6 to 12 months period immediately after an attack.

#### 5) Views of service users

Most participants were supportive of the trial, particularly if they had experienced several episodes of cellulitis in the past. Nevertheless, many concerns were documented and these will now be used when developing the participant information sheets to be used in the future.

#### 6) Views of investigators

Following this pilot study, the majority of investigators were willing to recruit for the future RCT. However, many were frustrated by the difficulties experienced in identifying patients and with the level of paperwork required for the ethics and R&D approval. Several felt that additional resources, or the provision of administrative support would make recruitment easier. One dermatologist reported a tension between the need to identify potential risk factors, such as toe-web maceration, and the desire to provide treatment advice once this had been identified. This has significant implications for the trial design as the treatment of potential risk factors prior to discharge may well impact on subsequent recurrence rates. If this is so, the estimated power of the trial could be reduced.

### Implications for the future trial

Many issues relating to the conduct and management of the future RCT have been raised by this pilot study. Table 3 summarises the key discussion points raised, along with the resulting conclusions. The overriding concern was to design a pragmatic study, that reflected actual practice as far as possible.

Two randomised controlled trials looking at the prevention of cellulitis of the leg have since been funded (one by Action Medical Research and one by the BUPA Foundation – ISRCTN 34716921). These funding applications were greatly enhanced by the preliminary work described in this report and we were in a position to make informed responses to the referees' comments as a result of it.

## Conclusion

This preliminary work has proved invaluable in highlighting some of the key issues to be addressed by an RCT of prophylactic antibiotics for the prevention of recurrent cellulitis. For a new clinical research network such as the UK DCTN, this has also been an opportunity to establish standard operating procedures and to train investigators in the conduct of clinical trials.

## Competing interests

The author(s) declare that they have no competing interests.

## Authors' contributions

KT (University of Nottingham) was the chief investigator, wrote the study protocol and paper, assisted investigators in obtaining ethics and R&D approvals and supervised the management of the study.

NC (Cumberland Infirmary) submitted the trial suggestion to the UK DCTN, helped to write the study protocol and reviewed the manuscript.

BS (University of Nottingham) co-ordinated the day-to-day management of the study and helped to write the paper.

DS (Bristol Royal Infirmary) recruited participants into the study, gave suggestions for future recruitment strategies and reviewed the manuscript.

SM (MRC Clinical Trials Unit) helped to develop the study protocol and commented on the manuscript.

AN (MRC Clinical Trials Unit) helped to develop the study protocol, provided statistical advice and commented on the manuscript.

NR helped to develop the study protocol and commented on the manuscript.

HW helped to develop the study protocol and commented on the manuscript.

All authors read and approved the final manuscript.

## Funding

This work was kindly supported by a grant from the British Skin Foundation. The central co-ordinating centre for the UK DCTN is supported by a grant from the National Co-ordinating Centre for Research Capacity Development.
